# Role of febuxostat in retarding progression of diabetic kidney disease with asymptomatic hyperuricemia: A 6-months open-label, randomized controlled trial

**DOI:** 10.17179/excli2018-1256

**Published:** 2018-06-13

**Authors:** Mohd Noor Azreey Mukri, Wei-Yen Kong, Ruslinda Mustafar, Syahrul Sazliyana Shaharir, Shamsul Azhar Shah, Abdul Halim Abdul Gafor, Rozita Mohd, Rizna Abdul Cader, Lydia Kamaruzaman

**Affiliations:** 1 Nephrology Unit, Department of Medicine, National University of Malaysia, Jalan Yaakob Latif, Bandar Tun Razak, Cheras 56000, Kuala Lumpur, Malaysia; 2 Rheumatology Unit, Department of Medicine, National University of Malaysia, Jalan Yaakob Latif, Bandar Tun Razak, Cheras 56000, Kuala Lumpur, Malaysia; 3 Department of Community Health, UKM Medical Molecular Biology Institute, Jalan Yaakob Latif, Bandar Tun Razak, Cheras 56000, Kuala Lumpur, Malaysia

**Keywords:** febuxostat, proteinuria, chronic kidney failure, HbA1c, hyperuricemia

## Abstract

**Introduction:** Hyperuricemia is associated with chronic kidney disease (CKD) progression and poor cardiovascular outcomes. We studied the effect of febuxostat on estimated glomerular filtration rate (eGFR), proteinuria and monitored the safety profile of the medication.

**Material and Methods: **This is a prospective open-label, randomized study in CKD stage 3 and 4 patients with diabetic nephropathy and asymptomatic hyperuricemia. Patients were randomized into febuxostat 40 mg daily and no treatment group using block randomization method and were followed up for 6 months. Their usual care for diabetes mellitus, hypertension and dyslipidemia were continued in the study. Blood and urine investigations were monitored at baseline, 3 months and 6 months.

**Results:** The eGFR in febuxostat group was stabilized at 6 months with no significant reduction [26.2 (IQR 14.30) at baseline to 26.3 (IQR 15.2) ml/min/1.73 m^2^]. Whereas, there was a significant reduction of the eGFR in no treatment group from 28.2 (IQR 17.9) to 27.6 (IQR 19.3) ml/min/1.73 m^2^ (*p* value < 0.01). We found the HbA1c (glycosylated hemoglobin) was significantly increased in febuxostat group from 7.2 ± 0.5 % at baseline to 7.6 ± 1.4 at 6 months (*p* value 0.04) but no significant change of HbA1c in the no treatment group. Proteinuria level was unchanged in both groups. The commonest adverse event was joint pain.

**Conclusions:** Febuxostat was able to preserve eGFR in CKD patients with diabetic nephropathy and this effect was beyond glycemic control. Increment of HbA1c level in febuxostat group needs further larger trials.

## Introduction

Hyperuricemia is defined as a serum uric acid level of more than 404 µmol/L (6.8 mg/dl) and has been considered as one of the risk factors for the onset and development of CKD, progression of renal dysfunction and cardiovascular mortality (Mende, 2015[19]; Prasad Sah and Qing, 2015[22]). There were many large observational studies confirmed the association between the incident (Chonchol et al., 2007[3]; Weiner et al., 2008[30]) and progression of CKD (Domrongkitchaiporn et al., 2005[4]; Obermayr et al., 2008[21]; Wang et al., 2011[29]). Nevertheless, despite known association of hyperuricemia and CKD, the treatment and management of asymptomatic hyperuricemia remain debatable. In Kidney Disease Improving Global Outcomes (KDIGO) CKD guidelines 2013, there was no strong recommendation on the management of asymptomatic hyperuricemia (KDIGO, 2012[15]). However, in the revised guideline 2012 in Japan, it was recommended to treat asymptomatic hyperuricemia of ≥ 475 µmol/l (8.0 mg/dl) (Yamanaka, 2012[34]). They suggested allopurinol as the first line treatment for hyperuricemia regardless whether asymptomatic, presence of urolithiasis, concomitant CKD or cardiovascular risk factors. They emphasized the dose needs to be adjusted as per renal function. 

Nevertheless, the use of allopurinol is limited by the risk of life threatening adverse effects such as Steven Johnson syndrome or toxic epidermal necrosis. These effects have been linked with HLA B 58:01 that is commonly related to Han Chinese population and is markedly predominant in South East Asia region (Lee et al., 2013[17]). Therefore, febuxostat, a novel non-purine selective xanthine oxidase inhibitor is a good alternative in the management of hyperuricemia. Renal adjustment is also not required in CKD patients with mild to moderately reduced eGFR as it is metabolized mainly by glucuronidation and oxidation in the liver and well excreted by both urinary and fecal routes (Garcia-Valladares et al., 2011[6]; Whelton et al., 2013[31]). It has been shown to be safe and efficient in CKD without major adverse events like allopurinol (Akimoto et al., 2014[1]; Ishikawa et al., 2014[12]). Sircar et al. in 2015 demonstrated that febuxostat slowed the eGFR decline in CKD patients with asymptomatic hyperuricemia regardless of any causes of the CKD (Sircar et al., 2015[25]).

The cut-off point for serum uric acid in asymptomatic hyperuricemia treatment was mentioned in one of the guidelines published in Japan with level of > 475 µmol/L (Yamanaka, 2012[34]) with only two previous studies had used level ranged from 416 to 430 µmol/L (Sircar et al., 2015[25]; Goicoechea et al., 2015[7]). Hence, this study is designed to assess the efficacy of febuxostat in preventing the progression of CKD in specific group of patients with diabetic nephropathy and good diabetic control (HbA1 < 8.0 %) (Ministry of Health Malaysia, 2015[20]) with asymptomatic hyperuricemia (≥ 400 µmol/L) and also monitoring the adverse events of the drug. This study also aimed to highlight the safety and efficacy of febuxostat in the treatment of asymptomatic hyperuricemia. 

## Material and Methods

### Study design

This single-centre open-labelled randomized study was conducted in Hospital Canselor Tuanku Muhriz, Universiti Kebangsaan Malaysia Medical Centre (UKMMC), Kuala Lumpur for a 6 months period with follow up. The study's protocol was approved by Research Ethics Committee, Universiti Kebangsaan Malaysia in accordance to the International Conference of Harmonisation Good Clinical Practice Guideline (Project Code: FF-2017-309). The Chronic Kidney Disease Epidemiology Collaboration (CKD EPI) study equation was used for estimation of GFR throughout the study period.

The primary objective was to determine the effect of febuxostat in slowing the eGFR decline in CKD stage 3 and 4 patients with diabetic nephropathy and asymptomatic hyperuricemia. Meanwhile the secondary objectives were to determine the effect of febuxostat in reducing proteinuria and to monitor adverse effects of febuxostat such as cardiovascular events i.e. myocardial infarction, stroke, heart failure and death.

All eligible patients from both gender aged 18 to 75 years old with CKD (eGFR ranged from 15 to 60 ml/min/1.72 m^2^) were included. To avoid poor glycemic control as the confounding factor of decline in eGFR, only patients with good glycemic control of Diabetes Mellitus (HbA1c < 8.0 %) were recruited. All patients must have asymptomatic hyperuricemia with serum uric acid level ≥ 400 µmol/L, never on any urate lowering therapy previously and has been on optimal tolerated dose of anti-proteinuric agents (Angiotensin Converting Enzyme inhibitor (ACEi) / Angiotensin Receptor Blocker (ARB) / calcium channel blocker). Patients who were on renal replacement therapy, history of allergy to febuxostat, medical history of heart failure, gouty arthritis and chronic liver diseases of all etiologies were excluded in this study.

Patients were allocated using block randomization into two groups; treatment (febuxostat 40 mg daily) and no treatment. Written consent were obtained at the first consultation. Standardized treatment for diabetes mellitus, hypertension and dyslipidemia were continued in the study. The previous doses of anti-hypertensive including all Renin Angiotensin Aldosterone System (RAAS) blockade agents (either ACE inhibitor or ARB) or Calcium channel blockers were continued and adjusted per patient's blood pressure control. Diuretics will be given as required. In treatment group, febuxostat was continued for 6 months with careful monitoring and any adverse reactions were recorded. If joint pains developed after the treatment, patient will be assessed and treated with short course of colchicine. Patients were reviewed at baseline, 1 month (via telephone consultation), 3 months and 6 months (clinic follow up). Every patient had at least 3 renal and liver function tests, urine protein creatinine index, full blood counts and HbA1 throughout the study period. 

A prior study by Sircar et al. (2015[25]) indicates that the difference in the response of matched pairs is normally distributed with standard deviation of 15. If the true difference in the mean response of both groups is 6.5, it is estimated that 85 pairs of subject is required in this study to reject the null hypothesis: the difference of eGFR decline in both groups is not significant with probability (power) of 0.8. The type I error probability of this test is 0.05. Therefore, a total number of 200 subjects will be recruited with equal number on both groups (100 subjects for each group) and including the 20 % of expected drop out from the study.

Randomization of patients recruited was based on block size of 4 or 8; either A (febuxostat) or B (no treatment), and proceeds by allocating random permutation of treatments within each block depending on number of patients per clinic visit (Efird, 2011[5]). Both patients and investigators were aware about the group allocation and medications given. Specific instructions were given to patients regarding possibility of any side effects and subsequent actions to be taken were also informed.

Data were analyzed using IBM SPSS Statistics Version 21 and based on an intention to treat analysis. Those who attended the first visit with at least 2 follow-up will be included in the analysis. All continuous variables were expressed as mean ± standard deviations for normally distributed data or median and interquartile range for non-normally distributed and categorical data described as proportion. All baseline variables in each group were compared to detect any variability. To determine differences between groups, Fisher exact or Pearson Chi Square test were used for all categorical data where applicable. For continuous data, independent *t*-test and paired *t-*test were used for normally distributed data whereas Mann Whitney or Wilcoxon Signed Rank test applied for not normally distributed continuous data.

## Results

**Figure 1:**
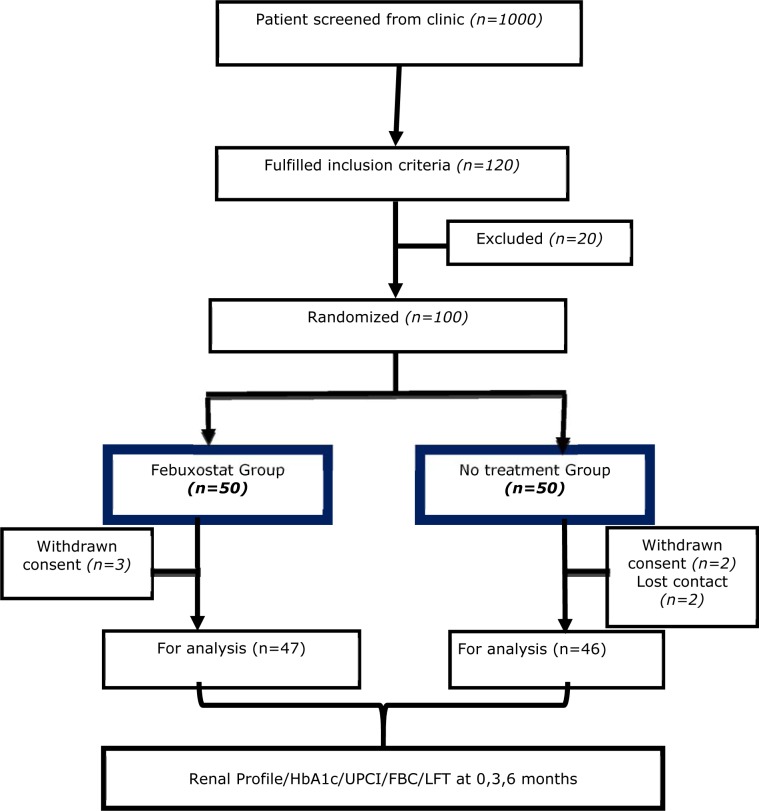
Flow diagram of single-centre trial evaluating febuxostat in comparison to no treatment group in CKD patients stage 3 and 4 with diabetic nephropathy and asymptomatic hyperuricemia

One thousand patients were screened initially from chronic kidney disease clinic list (Figure 1(Fig. 1)) and only one hundred and twenty patients fulfilled inclusion criteria. Subsequently only 100 patients randomized into the two groups: febuxostat and no treatment. Eventually, 47 patients were allocated in the febuxostat group and 46 patients in the no treatment group. Three patients in febuxostat group and one patient from no treatment group had dropped out from the study before completion and they were included in analysis.

All patients were recruited from July 2017 till October 2017 and the follow-up continued till end of March 2018. Baseline characteristics of the patients were illustrated in Table 1(Tab. 1). There were no significant differences of all variable across the two groups except for baseline HbA1c, height and body mass index (BMI). Most patients in both groups needed at least 3 combinations of anti-hypertensive medications. Calcium channel blockers (such as amlodipine and felodipine) was the most common drug prescribed followed by ACE inhibitors/ARB and diuretics as mentioned in Table 2(Tab. 2). For anti-diabetic agents (Table 2(Tab. 2)), insulin was the most common drug used. 

### ACE-Angiotensin converting enzyme-Angiotensin Receptor Blocker, CCB-Calcium Channel Blockers, DPP4-Dipeptyl Peptidase 4, BIDS- Basal Insulin Daytime Sulfonylurea

**Figure 2:**
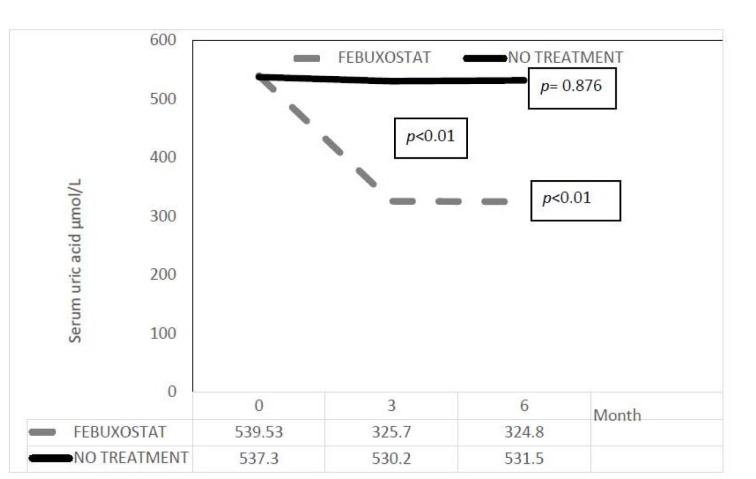
Changes in uric acid level

As expected uric acid level decreased significantly in febuxostat group as shown in Figure 2(Fig. 2) with the mean level reduced from 542.5 ± 101.2 µmol/L at baseline to 331.6 ± 139.8 µmol/L at 6 months (*p* value <0.01). Whereas in the no treatment group, the mean level was not significantly changed from 540.7 ± 72.0 to 538.7 ± 87.1 µmol/L. The serum uric acid difference in two groups at 6 months was also significant at -207.1 (-255.2 to -159) µmol/L with *p* value of < 0.01. 

**Figure 3:**
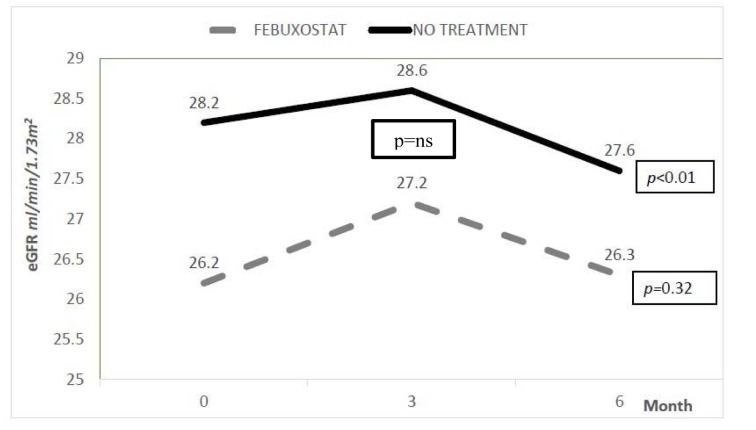
Changes in eGFR *ns= not significant

**Figure 4:**
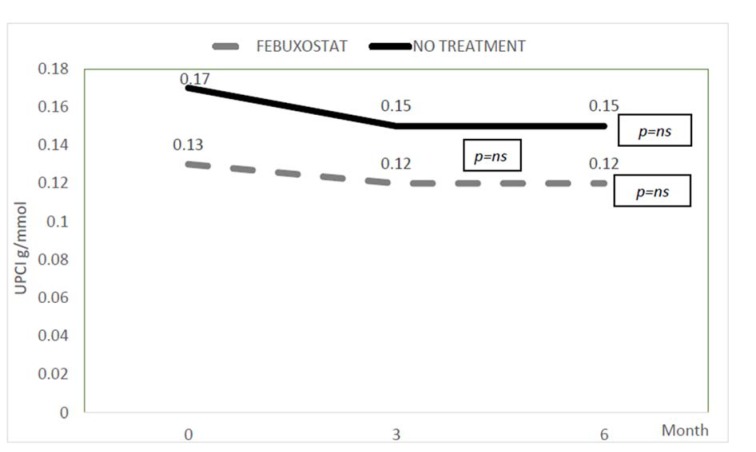
Changes in urine protein creatinine index *ns= not significant

The changes in eGFR and urine protein creatinine index in both febuxostat and no treatment groups were shown in Figure 3(Fig. 3) and 4(Fig. 4). The eGFR value (median) in febuxostat group was stabilized with no significant reduction from 26.2 (14.30) ml/min/1.73 m^2 ^at baseline to 26.3 (15.2) ml/min/1.73 m^2^ at 6 months. Meanwhile, in no treatment group, the eGFR was significantly reduced from 28.2 (19.8) ml/min/1.73 m^2 ^to 27.6 (20) ml/min/1.73 m^2^ (*p* value < 0.01). However, between these two groups, there was no significant difference in eGFR at 6 months of treatment. The urine protein creatinine index also did not show any significant changes in both groups with no significant difference between two groups at 6 months.

**Figure 5:**
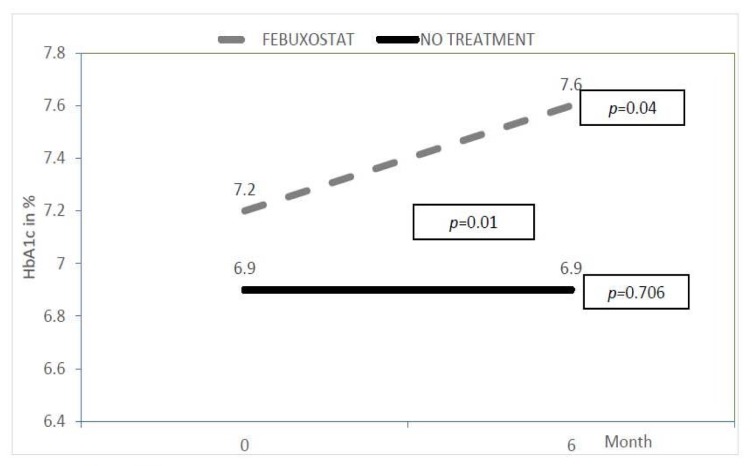
Changes in HbA1c

In addition, we found baseline HbA1c for the febuxostat group was significantly higher (7.2 ± 0.5 %) compared to the no treatment group (6.9 ± 0.7 %) with *p* value of 0.01. HbA1c in the febuxostat group continued to increase significantly at the end of study (Figure 5(Fig. 5)), from 7.2 ± 0.5 % to 7.6 ± 1.4 % (*p* value = 0.04). However, the HbA1c was not statistically changed in the no treatment group. Furthermore, the mean difference of HbA1c was also significant in between the two groups at 6 months with value of 0.63 % (0.14-1.11) and the *p* value was 0.01.

The most common adverse event observed in the study (Table 3(Tab. 3)) was joint pain and it was reported in 7 patients (15 %) taking febuxostat while none happened in the no treatment group. The next common adverse reaction was cardiovascular events; 4 patients (9 %) in febuxostat and 1 patient (2 %) in the no treatment group. Among four patients in the febuxostat group, one patient died due to recurrent stroke and had myocardial infarction, two patients developed fluid overload and one case developed complete heart block. One patient in the no treatment group had fluid overload due to worsening kidney function. There were two patients, who developed acute kidney injury in both groups, but their occurrences were most likely due to other factors such as fenofibrate (in febuxostat group) and NSAIDs abuse (in no treatment group) and required one episode of urgent hemodialysis). The least common side effects are gastrointestinal discomfort (one patient in febuxostat) and one case of transaminitis in no treatment group as patient took concomitant chlorophyll supplement and it resolved after discontinuation.

## Discussion

The present study showed that febuxostat, when given to CKD patients with diabetic nephropathy and asymptomatic hyperuricemia, decreases the uric acid and preserved the eGFR. However, the eGFR difference was not significant between the two groups. In addition, febuxostat did not reduce proteinuria level. Furthermore, the adverse effects such as joint pain and cardiovascular events need to be monitored carefully.

The majority of patients with hyperuricemia are asymptomatic and prevalence has been increasing over past decades especially in Asian countries (Smith, 2015[26]). High uric acid level has been previously studied and linked to the pathogenesis, development and progression of metabolic, renal and cardiovascular disease including hypertension, stroke and ischemic heart disease (Hosoya and Nishio, 2016[10]; Sharaf El Din et al., 2017[24]). Recent study in Spain found that hyperuricemia was associated with higher mortality and major cardiovascular event rates following acute coronary syndrome (Lopez-Pineda et al., 2018[18]). Furthermore, increasing level of uric acid in certain medical conditions such as acute myeloid leukemia, cardiac surgery or radiocontrast nephropathy may lead to acute kidney injury and hence at risk for development of chronic kidney disease (Hahn et al., 2017[8]). Another study in Taiwan has shown that higher uric acid level is associated with a significant rapid decline in eGFR particularly in patients without proteinuria and they are at considerable risk of kidney failure (Tsai et al., 2017[27]). In previous Modification of Diet in Renal Disease (MDRD) study, proteinuria has been described to be a major predictor in rapid eGFR decline in most patients (Hunsicker et al., 1997[11]) and it has been supported by a prospective observational study by Zoppini et al. which included type 2 diabetic patients and the strongest predictor of annual GFR decline was albuminuria followed by older age, hypertension, insulin treatment and lower baseline eGFR (Zoppini et al., 2012[35]).

Hyperuricemia has been defined as accumulation of serum uric acid beyond its solubility point in water and develops because of uric acid over production, under secretion or both (Jalal et al., 2013[13]; Wright et al., 2010[33]). The underlying pathogenesis of hyperuricemia leading to kidney injury includes deposition of intra-luminal crystal in the collecting duct of the nephron as uric acid is less soluble than urate (Jalal et al., 2013[13]). The uric acid crystals formed will adhere to the surface of the renal epithelial cells and induce an acute inflammatory response and hence will lead to reduce GFR (Jalal et al., 2013[13]). Uric acid has been shown to activate cytoplasmic phospholipase A_2 _and the inflammatory transcription nuclear factor κ B (NF - κB), leading to inhibition of proximal tubular cellular proliferation in vitro (Han et al., 2007[9]). The sequelae of raising uric acid will also lead to systemic cytokine production such as tumour necrosis factor α (TNF-α) and local expression of chemokines such as monocyte chemotactic protein 1 (MCP-1) in kidney and cyclooxygenase 2 (COX-2) in blood vessels (Jalal et al., 2013[13]; Kang and Nakagawa, 2005[14]). The other mechanism was stimulation of the renin-angiotensin system and inhibit the release of endothelial nitric oxide, leading to renal vasoconstriction and hypertension (Han et al., 2007[9]). All these mechanisms will eventually lead interstitial inflammation and progression of kidney injury.

Our study findings were consistent with Sircar et al. in 2015 who also demonstrated eGFR stabilization at 6 months but there was a different profile of adverse events found in our study. Sircar et al., had shown that the baseline eGFR in febuxostat group was stable from 31.5 ± 13.6 to 33.7 ± 16.6 mL/min/1.73 m^2^ at 6 months while mean eGFR significantly reduced in the placebo group from 32.6 ± 11.6 to 28.2 ± 11.5 mL/min/1.73 m^2 ^(Sircar et al., 2015[25]). The inter-group difference in eGFR was significant at 6 months with no serious adverse events (Sircar et al., 2015[25]). Another systematic review and meta-analysis showed that febuxostat had an anti-proteinuric effect with no difference in eGFR and serum creatinine level but limited by the amount of studies included and shorter duration of study (Kim et al., 2017[16]). 

In our study, the baseline HbA1c level was higher in the febuxostat group and the value was significantly increased at the end of our study compared to the no treatment group. Higher HbA1c level had been previously described as one of the risk factor for eGFR decline and CKD progression in diabetic nephropathy patients (Rossing et al., 2004[23]). An increase in HbA1c variability had been described as a signal of previous poor glycemic control and hence may trigger metabolic phenomenon where the diabetic complications particularly diabetic nephropathy continues despite good sugar control later on (Cheng et al., 2014[2]). The variability of HbA1c may activate the oxidative stress and consequently worsen the diabetic complications such as diabetic nephropathy, peripheral neuropathy and peripheral vascular disease (Cheng et al., 2014[2]). Interestingly the HbA1c level was increased with febuxostat used and there was no previous study linked the glycemic control to the drug. Therefore the exact explanation for this finding remains unanswered. We could postulate, might be there was a change of patients' diet due to their awareness that they were on study medication. In addition, febuxostat did not reduce the proteinuria level. Despite the significant increase in HbA1c and unchanged proteinuria level, the eGFR remained stable in the febuxostat group. Therefore, the possible renoprotective effect of febuxostat was believed to be beyond the glycemic control and proteinuria itself. The effect may be due to significant reduction of uric acid and hence leads to decrease production of pro-inflammatory markers and less activation of renin angiotensin system. 

The Confirmation of Febuxostat in Reducing and Maintaining Serum Urate (CONFIRMS) trial has demonstrated the potential side effects of febuxostat with the most common was gastrointestinal disturbances and elevated liver transaminases (Garcia-Valladares et al., 2011[6]). However, the United States Food and Drug Administration (FDA) had released a warning in November 2017 regarding potential cardiovascular side effects and may be used with extra cautions (U.S. FDA, 2017[28]). Subsequently, a new study, the Cardiovascular Safety of Febuxostat or Allopurinol in Patients with Gout (CARES) trial published recently in March 2018, showed that febuxostat was non-inferior to allopurinol in the rates of cardiovascular events but all-cause mortality and cardiovascular mortality were higher with febuxostat (White et al., 2018[32]). In our study, the most common adverse event was joint pain and a trend of higher cardiovascular events in the febuxostat group and yet statistically it was not significant. It was not demonstrated by Sircar et al. previously and possibly the rate of cardiovascular events related to the background cardiovascular risk factors and history (i.e. recurrent stroke or ischemic heart disease) in this study. 

 The limitations of this study were a small number of patients (involved single centre) and short study duration (6 months). The eGFR values 6 months prior to recruitment were also not included in this study. Longer study duration and more number patients are needed to evaluate the efficacy of febuxostat. Double blinded process in randomization and possibly eGFR trend prior to clinical trial need to be included in future studies. 

## Conclusion

Preservation of eGFR in diabetic nephropathy patients with febuxostat needs further confirmation in the future larger and long-term studies. Despite this promising evidence on eGFR preservation, the use of febuxostat in diabetic nephropathy patients warrants further caution and close monitoring as it may increase their HbA1c and the potential risk of cardiovascular event.

## Acknowledgement

We would like to acknowledge the Pharmacy Department, UKM Medical Centre and Research Ethics Committee, The National University of Malaysia.

## Conflict of interest

The authors declare no conflict of interest in this clinical trial.

## Figures and Tables

**Table 1:**
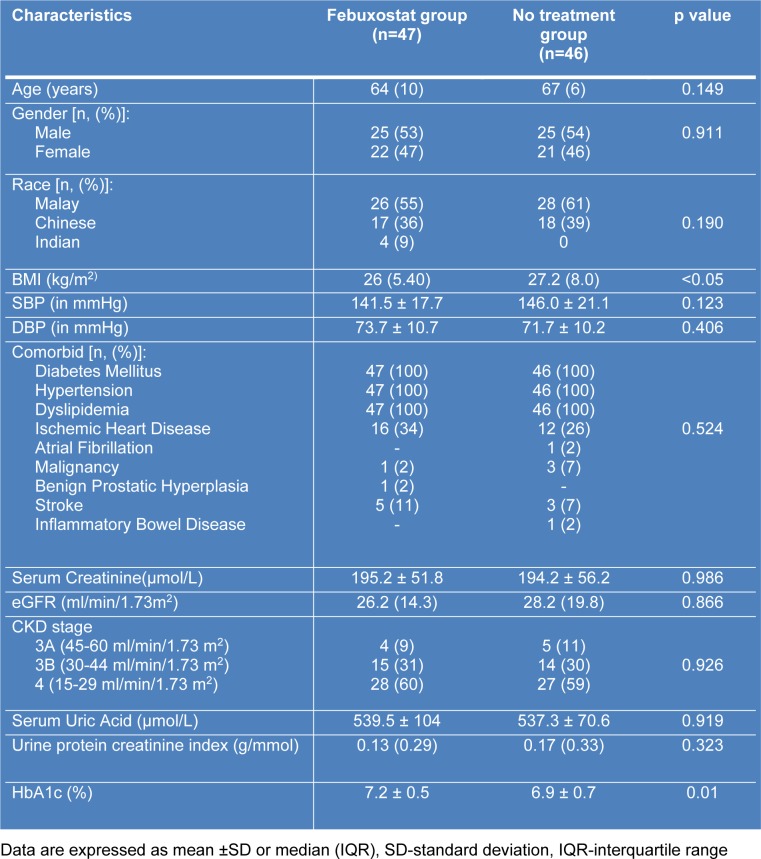
Baseline demographics and laboratory data

**Table 2:**
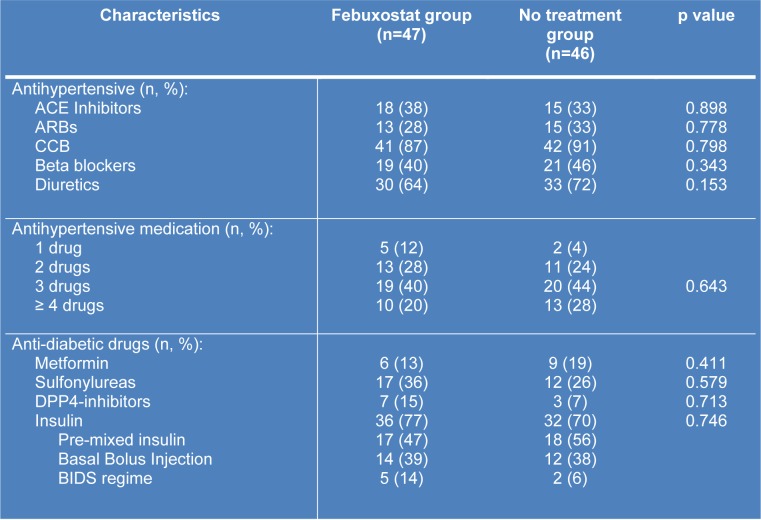
List of medications used

**Table 3:**
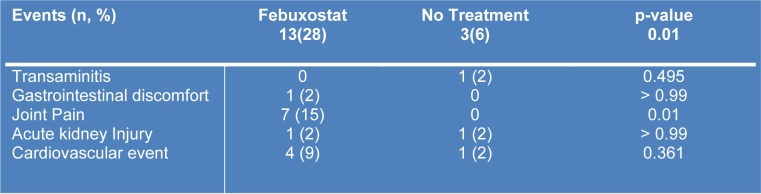
Adverse events throughout the study period
